# Association between metformin and the risk of gastric cancer in patients with type 2 diabetes mellitus: a meta-analysis of cohort studies

**DOI:** 10.18632/oncotarget.16973

**Published:** 2017-04-08

**Authors:** Xue-Liang Zhou, Wen-Hua Xue, Xian-Fei Ding, Li-Feng Li, Meng-Meng Dou, Wei-Jie Zhang, Zhuan Lv, Zhi-Rui Fan, Jie Zhao, Liu-Xing Wang

**Affiliations:** ^1^ Department of Oncology, The First Affiliated Hospital of Zhengzhou University, Zhengzhou, Henan, China; ^2^ Department of Pharmacy, The First Affiliated Hospital of Zhengzhou University, Zhengzhou, Henan, China; ^3^ Department of General ICU, The First Affiliated Hospital of Zhengzhou University, Zhengzhou, Henan, China; ^4^ Department of Integrated Traditional and Western Medicine, The First Affiliated Hospital of Zhengzhou University, Zhengzhou, Henan, China

**Keywords:** gastric cancer, metformin, type 2 diabetes mellitus, risk, meta-analysis

## Abstract

**Objectives:**

The objective of this study was to evaluate the association between metformin therapy and the incidence of gastric cancer (GC) in patients with type 2 diabetes mellitus (T2DM).

**Methods:**

We systemically searched the following databases for studies published between the databases’ dates of inception and Nov. 2016: PubMed, Embase, the Cochrane Library, the Web of Science, and the China National Knowledge Infrastructure (CNKI). Hazard ratios (*HR*)and corresponding 95% confidence intervals (*CIs*) for the association between metformin therapy and the incidence of GC in patients with T2DM were the outcome measures assessed in this study. STATA 12.0 (Stata Corporation, College Station, Texas, USA) was used to conduct the statistical analysis.

**Results:**

A total of seven cohort studies including 591,077 patients met all the criteria for inclusion in the analysis. Our data showed that metformin therapy was associated with a significantly lower incidence of GC in patients with T2DM than other types of therapy (*HR*=0.763, 95% *CI*: 0.642˜0.905). Subgroup analysis showed that patients living in Taiwan benefitted more from metformin therapy than patients living in any other region, as metformin significantly decreased the risk of GC in patients living in Taiwan but did not significantly decrease the risk of GC in patients living in other regions (*HR*=0.514, 95% *CI*: 0.384-0.688). The results of the present analysis support the idea that metformin facilitates reductions in the risk of T2DM-related GC.

**Conclusions:**

The risk of GC among patients with T2DM is lower in patients receiving metformin therapy than in patients not receiving metformin therapy.

## INTRODUCTION

Gastric cancer (GC) is a leading cause of cancer-related mortality worldwide. Approximately 951,600 new cases of stomach cancer and 723,100 stomach cancer-related deaths occurred in 2012. Eastern Asia, Eastern Europe, and South America have the highest incidences of GC [1]. Currently, the combination of chemotherapy and surgery is the most effective means of preventing GC recurrence and progression. Most patients with GC are diagnosed at advanced disease stages, and the overall 5-year survival rate for patients with resectable GC remains low at 10-30% despite advances in the surgeries and clinical therapies used to treat the disease [2]. Patients who present with inoperable, advanced or metastatic disease require palliative treatment; however, early disease detection is more common in Asia than in other regions [3]. Thus, given the shortcomings of current GC infusion chemotherapy drugs, the inconsistencies in the efficacies of specific drugs and the severity of the side effects associated with specific drugs, identifying safe and effective treatments for GC has become an important issue with respect to the treatment of the disease.

Type 2 diabetes mellitus (T2DM) is a prevalent disease associated with a large global public health burden [4]. The prevalence of diabetes mellitus has increased substantially in recent years and is believed to be associated with an increased risk of many cancers [5]. Considerable numbers of epidemiological studies [6] and systematic reviews have demonstrated the existence of positive associations between diabetes mellitus and the risks of biliary tract cancer [7], liver cancer [8], kidney cancer [9], colon cancer [10], pancreas cancer [11], bladder cancer [12], ovarian cancer [13], breast cancer [14], non-Hodgkin lymphoma [15], prostate cancer [16], and lung cancer [17].

Metformin is a first-line treatment for T2DM. Many basic *in vitro* and *in vivo* studies have shown that metformin can inhibit the growth of GC [18], esophageal cancer [19], colon cancer, breast cancer, liver cancer and other tumors [20–23]. Hypoxia inducible factor 1α (HIF1α) and glucose metabolism can change the tumor microenvironment [24], and HIF1α plays a critical role in regulating tumor angiogenesis in response to hypoxia [25]. When cells are hypoxic, the PI3k/Akt/HIF1α signaling pathway is activated to regulate tumor glucose metabolism [26]. Metformin may inhibit gastric cancer development and progression by inhibiting HIF1α/PKM2 signaling [27]. The findings of epidemiological studies indicate that patients with T2DM who are treated with metformin have a lower risk of GC than patients who are not treated with metformin. However, the results of some studies [28, 29] indicate that metformin cannot reduce the risk of GC in patients with T2DM. Therefore, whether metformin can reduce the incidence of GC in patients with T2DM is a controversial subject. Thus, we performed a meta-analysis to examine the effects of metformin treatment on the risk of GC among patients with T2DM. The findings of this analysis may provide clinicians with new ideas regarding possible clinical treatments for GC.

## RESULTS

**Figure 1:**
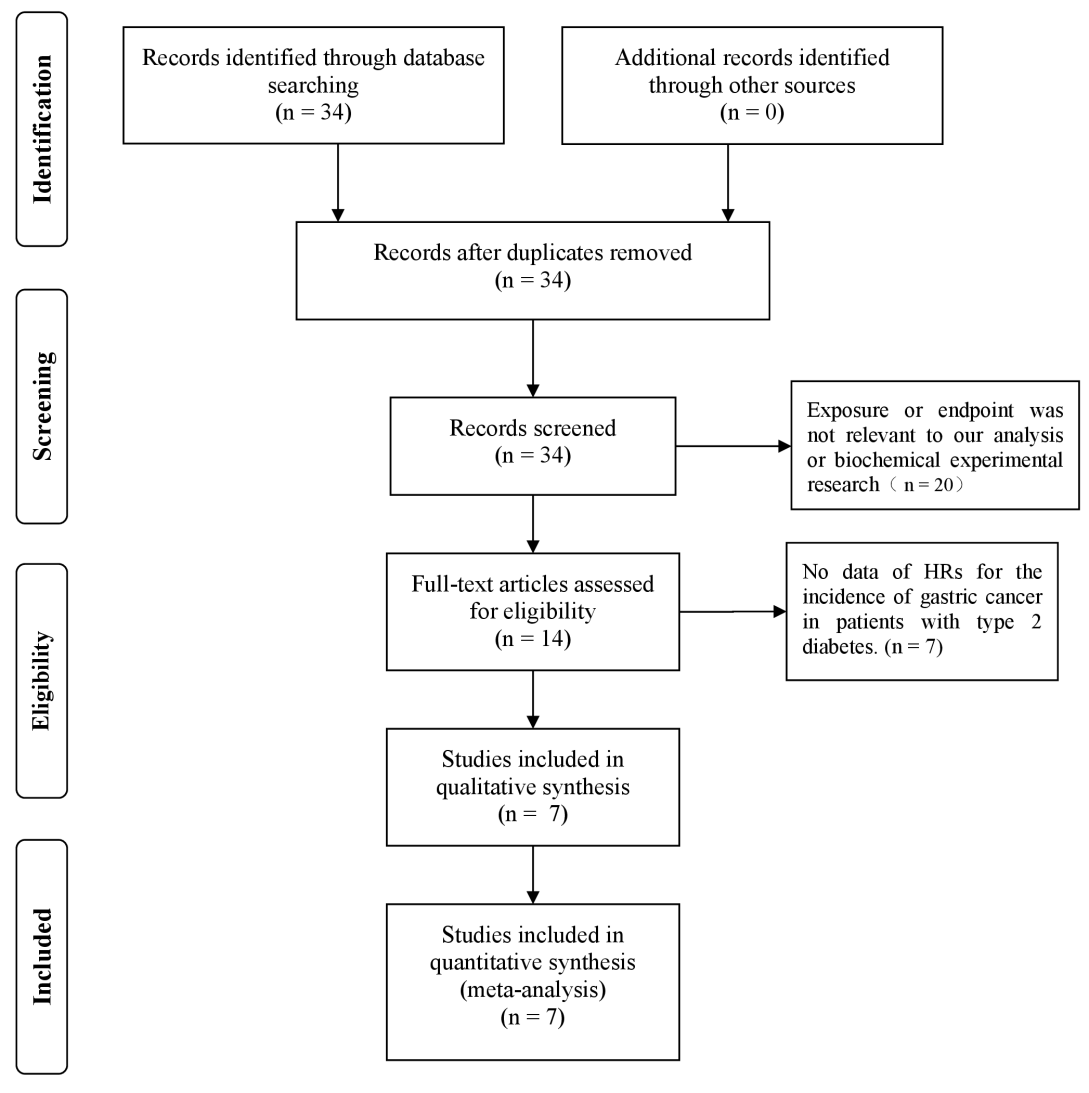
Flow diagram of the process by which the studies included in the analysis were selected

We initially identified 34 potentially eligible studies by title and abstract screening but excluded 20 of them because their exposures or endpoints were not relevant to our analysis or because they were biochemical experimental studies. After reviewing the full texts of the remaining 14 studies in detail, we excluded 7 studies because they did not assess the relationship between metformin and the indicated outcomes of interest. Thus, 7 studies (591,077 patients) [28–33] were ultimately included in our final analysis. A flow chart depicting the process by which these studies were selected is presented in Figure 1.

Data pertaining to the first author, year of publication, study region, study design, number of patients, treatment groups, comparators, and controlled variables are reported in Table 1. Of the studies included in the analysis, the majority were conducted in Asia. Specifically, 4 studies were conducted in China, and 1 study was conducted in Korea. Only 2 studies were conducted in Europe.

**Table 1:** Characteristics of studies included in the Meta-analysis

First author	Year of publication	Study region	Study design	Number of patients (case/control)	Age of patients (years)	Treatment Group	Comparators	Year of study conducted	HR (95%CI) of incidence	Controlled variables/notes
Valent, F	2015	Italy	RC study	63119/75402	20-94	Metformin	No Metformin	2002-2014	0.990 (0.986–0.994)	Age, sex, at start of observation (years), time-dependent variable and total number of prescriptions of all the other drugs
Ruiter, R	2012	Netherland	RC study	52698/32591	≥18	Metformin	Sulfonylurea	1998-2008	0.83 (0.76–0.90)	Age, sex, year in which the first OGLD prescription, number of unique drugs used in the year, and number of hospitalizations in the year before the start of the OGLD
Lee, M. S	2011	Taiwan	PC study	11215/4197	≥20	Metformin	No Metformin	2000-2007	1.41 (0.42-4.73)	Age, sex, other oral anti-hyperglycemic medication, Charlsoncomorbidity index score,
Kim, Y. I	2014	Korean	RC study	26690/6228	All ages	Metformin	No Metformin	2004-2010	0.73 (0.53–1.01)	Age, sex, residential area, and other anti-diabetic drug use
Hsieh, M. C1	2012	Taiwan	RC study	3963/6072	≥20	Metformin	Sulfonylurea	2000-2008	0.567 (0.340-0.946)	Age, sex
Hsieh, M. C2	2012	Taiwan	RC study	3963/751	≥20	Metformin	Insulin	2000-2008	0.541 (0.230-1.276)	Age, sex
Tseng, C. H.	2016	Taiwan	RC study	287971/16217	25-74	Metformin	No Metformin	1999-2011	0.448 (0.359-0.558)	NA

Abbreviations: NA: information not available; RC: retrospective cohort; PC: prospective cohort.

The quality of the eligible studies was assessed by the Newcastle-Ottawa Quality Assessment Scale (NOS), as recommended by the Cochrane Non-Randomized Studies Methods Working Group [34]. This scale rates studies based on the following three parameters: selection, comparability and outcome. Studies can receive a maximum possible score of nine stars and are graded as follows: studies receiving < 5 stars are rated as low-quality studies, and studies receiving > 6 stars are rated as high-quality studies. The quality assessment was performed by two investigators (Xianfei Ding, Mengmeng Dou), and any disagreements regarding study quality were resolved by Xueliang Zhou. The NOS used to assess the quality of the seven cohort studies included in the analysis is shown in Table 2.

**Table 2:** The Newcastle-Ottawa Scale (NOS) for assessing the quality of cohort studies

studies	Selection			Comparability			Assessment of Outcome			Total Quality score
First author	Representativeness of Exposure arm(s)	Selection of the comparative arm(s)	Origin of exposure source	Demonstration that outcome of interest was not present at start of study	Studies controlling the most important factors	Studies controlling the other main factors	Assessment of outcome with independency	Adequacy of Follow up length (to assess outcome)	Lost to follow up acceptable (less than 10% and reported)	HR(95%CI) of OS
Valent, F	*	*	*	*	*		*	*	*	8
Ruiter, R	*	*	*	*	*		*	*		7
Lee, M. S	*	*	*	*	*		*	*		7
Kim, Y. I	*	*	*	*	*		*	*	*	8
Hsieh, M. C1	*	*	*	*	*		*	*		7
Hsieh, M. C2	*	*	*	*	*		*	*		7
Tseng, C. H.	*	*	*	*	*		*	*	*	8

**Figure 2:**
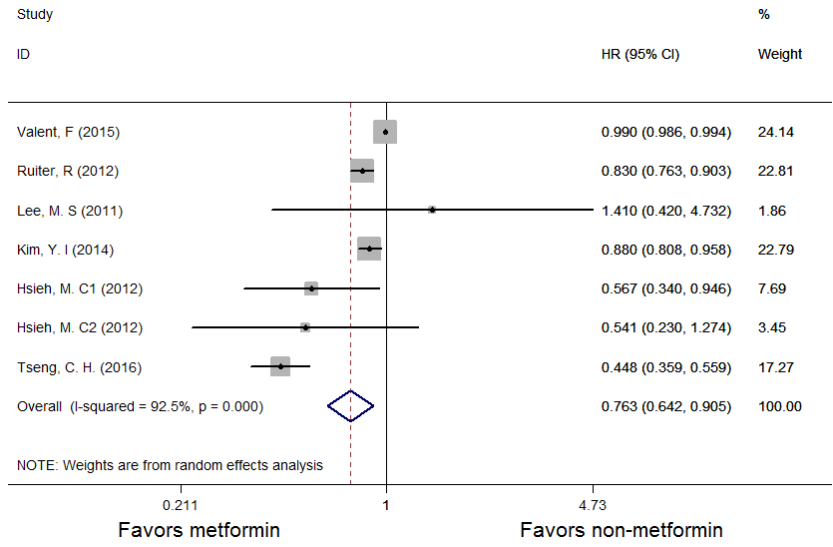
Forest plots of the association between metformin and the incidence of GC among patients with T2DM HR: hazard ratio; *CI*: confidence interval.

**Figure 3:**
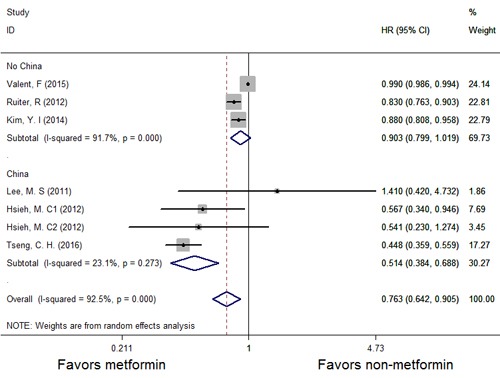
Forest plots illustrating the HRs and corresponding 95% CIs for the incidence of GC among patients with T2DM according to region (Taiwan and non-Taiwan)

A forest plot of the association between metformin therapy and the risk of GC is shown in Figure 2, and the results of a subgroup analysis evaluating the association between metformin therapy and the incidence of GC (*HR* with 95% *CI*) in specific regions (Taiwan and non-Taiwan) are shown in Figure 3. As shown in Figure 2, studies in which multivariate analyses were performed reported that the risk of GC was significantly decreased among patients receiving metformin therapy compared with patients receiving other therapies. However, our analysis uncovered strong evidence of the presence of significant heterogeneity among the studies (*P* = 0.000, *I*2 = 92.5%). Therefore, we used the random-effects model to conduct an analysis of the relationship between metformin therapy and the risk of GC across the studies. The summary *HR* for the relationship was 0.763 (95% *CI*: 0.642-0.905). To find the source of the heterogeneity, we performed an analysis in which the studies were organized into subgroups according to the geographic regions in which they were performed. We found that metformin therapy significantly decreased the risk of GC in the studies from Taiwan (*HR* = 0.514, 95% *CI*: 0.384-0.688) and that the heterogeneity among the indicated studies was low (*I*2 = 23.1%; *P* = 0.273); however, metformin therapy did not significantly decrease the risk of GC in the studies from regions other than Taiwan (*HR* = 0.903, 95% *CI*: 0.799-1.019), and the heterogeneity among the indicated studies was high (*I*2 = 91.7%; *P* = 0.000). Thus, the results of the subgroup analysis showed that the heterogeneity noted in this meta-analysis was mainly attributable to differences among studies performed within a specific study region.

**Figure 4:**
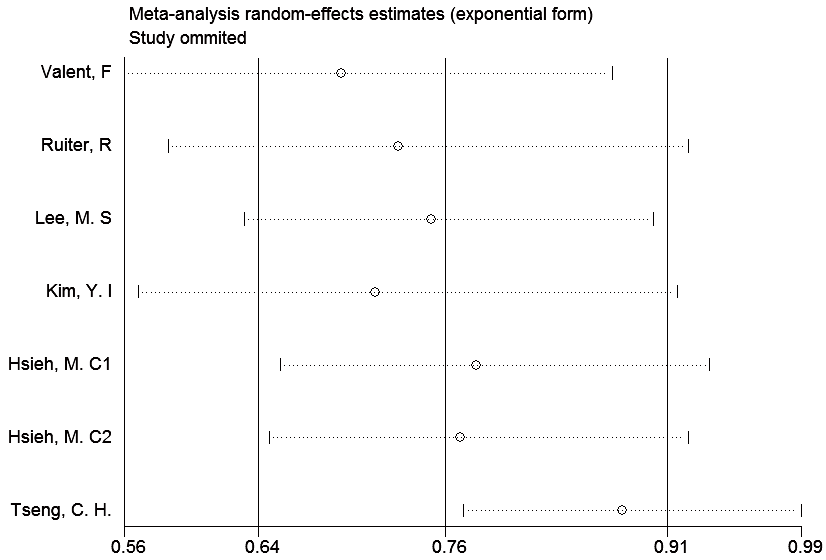
Sensitivity analysis of the incidence of GC among patients with T2DM

We conducted sensitivity analyses to verify the effect of each study on the overall estimate by omitting one study at a time and determining the overall estimate for the remaining studies. The results of the sensitivity analyses are shown in Figure 4. The results of the sensitivity analysis of the risk of cancer showed good consistency and indicated that omitting any one of the studies did not significantly affect the combined estimate and that the range of the results was fairly narrow. Thus, the results of our study indicated that the pooled estimate of our analysis was statistically robust.

As the number of studies included in the analysis was small ( < 10), we did not construct a funnel plot because it may not have detected publication bias [35].

## DISCUSSION

In this meta-analysis including 591,077 patients, we summarized the currently available evidence to determine the potential role of metformin in chemoprevention regimens intended to prevent GC. Metformin therapy was associated with a reduction in the risk of GC in patients with T2DM.

GC is the second most frequent cause of cancer-related death worldwide, and many factors and comorbidities, including hyperglycemia, Helicobacter pylori (H. pylori) infection, high salt intake, and medications, play a role in its development and progression [36]. T2DM was also recently shown to increase the risk of GC [5], and insulin resistance may be one of the causes of the increased risk of GC noted among patients with T2DM [37]. Therefore, preventative and personalized treatment regimens are the best options for reducing GC-related mortality rates [2]. Metformin, an oral anti-hyperglycemic agent and a member the biguanide family, is an effective, inexpensive and widely used first-line treatment for T2DM and is associated with fewer adverse reactions than other agents [38]. Given the close relationship between T2DM and the risk of gastric cancer [5, 39, 40], researchers have begun to pay attention to the relationship between the risk of cancer and metformin therapy in recent years. Some studies have shown that metformin therapy can reduce the risk of cancer in patients with T2DM [41, 42].

More and more evidence indicates that metformin may be useful as an anticancer drug. Numerous experimental studies have demonstrated that metformin has growth-inhibiting effects on breast [43], endometrial [44], lung [45], liver [46], gastric [18] and head and neck squamous cell carcinoma cells [47], as well as medullary thyroid [48] cancer cells. Regarding the mechanism underlying the anti-cancer effects of metformin, Kalender [49] showed that metformin disrupted mitochondrial energetics and inhibited mTORC1 kinase through an unknown mechanism, and Vazquez [50] showed that metformin reduces gluconeogenesis in the liver while promoting fat and muscle glucose uptake mainly through AMPK pathway activation to lower blood glucose levels. AMPK not only plays a key role in cell metabolism but also inhibits tumor cell proliferation and migration [51]. However, Chen [27] found metformin may inhibit gastric cancer by inhibiting HIF1α/PKM2 signaling. Therefore, the precise relationship between metformin therapy and the incidence of cancer, especially the effects of metformin therapy on GC, is unclear. Thus, we summarized the current data on the relationship between metformin and the risk of GC in this meta-analysis.

Increasing numbers of studies regarding the association between metformin and some types of cancer have been performed. Some meta-analysis have shown that metformin can reduce the risk of lung cancer [52], liver cancer [53], prostate cancer [54], colorectal cancer [55], and pancreatic cancer [56]. Our data showed that metformin significantly lowers the risk of GC in patients with T2DM compared with other therapies (*HR* = 0.763, 95% *CI*: 0.642-0.905). Moreover, our subgroup analysis showed that metformin significantly decreased the risk of GC in patients in Taiwan (*HR* = 0.514, 95% *CI*: 0.384-0.688) and that the amount of heterogeneity among the studies performed in that region was low (*I*2 = 23.1%; *P* = 0.273).

This meta-analysis had several advantages. First, sufficient resources were utilized to acquire data pertaining to the relationship between metformin therapy and the risk of GC, and these data were accurate. Second, sensitivity analysis of the relationship between metformin therapy and the incidence of GC showed that removing any one of the 7 studies from the meta-analysis did not significantly change the results of the analysis, indicating that the results are robust. Finally, the 7 studies and 591,077 patients included in the analysis had high NOS scores, indicating that the studies were of high quality and featured results applicable to the general population. Moreover, the criteria for study screening, inclusion and exclusion were strict, which ensured that the results of the meta-analysis are stable and reliable. However, the meta-analysis also had several limitations. First, the number of studies included in the analysis was small ( < 10); therefore, we did not generate a funnel plot and were thus unable detect publication bias. Second, we were unable to account for the influence of some important confounding factors that may have affected the results of our comprehensive analysis. Third, we were unable to obtain complete data regarding patient ages, metformin doses, and treatment durations, as well as data pertaining to potential confounders and risk factors.

In conclusion, metformin therapy is associated with a significantly lower risk of gastric cancer in patients with T2DM than other therapies. Subgroup analysis showed that metformin significantly reduced the risk of gastric cancer in patients with T2DM in Taiwan. The results of this study may serve as a theoretical basis for the treatment of tumors with metformin in the future. However, additional investigations, particularly blinded randomized controlled trials, are required to confirm the association noted herein.

## MATERIALS AND METHODS

### Inclusion criteria

The following studies were included in the meta-analysis: (1) original full-text articles published in English or Chinese that were designed to evaluate the association between metformin and the incidence of GC in patients with T2DM, (2) studies utilizing appropriate statistical analysis methods and possessing sufficient data, (3) studies with a cohort design comprising an observation group that received metformin therapy and a control group that received other antidiabetic drugs (such as sulfonylureas, thiazolidinediones, insulin or other drugs) and (4) studies reporting crude or adjusted estimates of the associations between exposures and outcomes (hazard ratios [*HRs*] and corresponding 95% confidence intervals [*CIs*]) or survival curves (Kaplan-Meier curves).

### Exclusion criteria

The following studies were excluded from the meta-analysis: (1) studies based on animal models, (2) studies based on cell models, (3) reviews, (4) studies lacking relevant data, and (5) studies lacking sufficient data for extraction.

### Search strategy

We conducted a comprehensive search of the following databases to identify studies published between the databases’ dates of inception and Nov. 2016: PubMed, Embase, the Cochrane Library, the Web of Science, and the Chinese National Knowledge Infrastructure (CNKI). The searches comprised a combination of medical subject heading (MESH) searches and text word searches for studies published in English and Chinese. The following key words were used for the searches: “Gastric Cancer” OR “Gastric carcinoma” OR “Gastric Neoplasm” OR “Stomach Cancer” OR “Stomach carcinoma” OR “Stomach Neoplasm”, “Metformin”. The reference lists of relevant articles and reviews were also scanned manually to identify additional relevant articles.

### Study selection

Two reviewers independently screened the titles and abstracts of the studies retrieved through the above searches and assessed the eligibility of each study by reading its full text. Any disagreements regarding study inclusion were resolved *via* consultation with a third reviewer.

### Data extraction

Two authors independently checked the accuracy of the data extracted from the articles that met the above inclusion criteria. Disagreements between the two authors regarding the data extracted from the studies were resolved by discussion and the achievement of consensus. We extracted the following information from each published article: (1) the title, author, date of publication and references; (2) the ages and ethnicities of the patients; (3) the prognostic indicators; and (4) the methodological quality assessment index.

### Statistical analyses

All statistical analyses were performed with STATA 12.0. HRs as effect sizes for morbidity were expressed as 95% CIs. *HRs* < 1 and 95% *CIs* that did not overlap with 1 were indicators that metformin therapy can decrease the risk of GC in patients with T2DM, while *HRs* > 1 were indicators that metformin therapy cannot decrease the risk of GC in patients with T2DM. Between-study heterogeneity was assessed using the Cochran Q test, whose significance level was *p* < 0.100, and the *I*2 test, whose significance level was *I*2 > 50%. Initial analyses were performed with a fixed-effects model, after which confirmatory analyses were performed with a random-effects model if significant heterogeneity was present.

## Abbreviations

GC: Gastric cancer
T2DM: Type 2 diabetes mellitus
HR: Hazard ratio
CI: Confidence interval
CNKI: China National Knowledge Infrastructure
MESH: Medical subject headings
NA: Information not available
RC: Retrospective cohort
PC: Prospective cohort.
